# Assessing the publishing priorities and preferences among STEM researchers at a large R1 institution

**DOI:** 10.1016/j.heliyon.2023.e16316

**Published:** 2023-05-15

**Authors:** Ibraheem Ali, Jason Burton, M. Wynn Tranfield

**Affiliations:** a University of California, Los Angeles, CA, USA; b University of California, Santa Cruz, CA, USA

**Keywords:** Research, Publishing preferences, Academic culture, Data management, Open access, Open science, Research assessment

## Abstract

The cost of academic publishing has increased substantially despite the ease with which information can be shared on the web. Open Access publishing is a key mechanism for amplifying research access, inclusivity, and impact. Despite this, shifting to a free-to-read publishing environment requires navigating complex barriers that vary by career status and publishing expectations. In this article, we investigate the motivations and preferences of researchers situated within our large research institution as a case study for publishing attitudes at similar institutions. We surveyed the publishing priorities and preferences of researchers at various career stages in STEM fields as they relate to openness, data practices, and assessment of research impact. Our results indicate that publishing preferences, data management experience and research impact assessment vary by career status and departmental approaches to promotion. We find that open access publishing is widely appreciated regardless of career status, but financial limitations and publishing expectations were common barriers to publishing in Open Access journals. Our findings shed light on publishing attitudes and preferences among researchers at a major R1 research institution, and offer insight into advocacy strategies that incentivize open access publishing.

## 1 Introduction

Research institutions and their associated libraries have been increasingly invested in understanding where researchers decide to publish and motivations behind those publishing decisions. These publishing choices fuel the moral economy of scholarly publishing, wherein researchers provide content, editorial expertise, and peer review without monetary compensation. Author choices also impact the overall openness of research in a broader sense. They determine how reproducible experiments are by virtue of the transparency of methods and associated data presented in the work. Publishing venues often dictate who may be excluded from accessing results based on the cost of subscriptions. Published work reflects research being done at a given institution, as well as where a library's collection may choose to optimize subscriptions and purchases. This study examines the scholarly publishing preferences and motivations of STEM researchers at a large R1 institution. A deeper understanding of researcher priorities will allow librarians and publishing advocates to provide more impactful outreach and negotiate meaningful compromises with publishers in support of open content.

Individual researcher journal selection motivations are still opaque. Authors may be motivated to choose select journals by disciplinary fit, cost of publishing, ease of submission, audience selection, or another unknown factor. Access costs for published content continues to outpace library budget allocations [[1], [2], [3], [4]]. Open Access publishing can alleviate subscription charges, but due to article processing charges are not “cost neutral” for institutions. Institutions have found themselves in search of innovative ways to promote author choices that may prove more sustainable long-term, and promote public access to research. Despite the article processing charges, supporting OA may be one of those strategies. Laakso et al. (2011) reported that in 2009, only 7.7% of peer-reviewed articles were open access [5]. This finding benchmarked a mandate from United States federal funding agencies that decried all publicly funded research must be publicly accessible [6]. By 2018 when the European Research Council launched Plan S, a similar mandate for publicly funded research, Science-Metrix reported that more than half of all peer-reviewed articles were available through various OA channels, including hybrid journals, OA journals, preprint servers, and post-print servers [7].

This rise in OA journal publishing has led to the creation of a new wave of OA journals with questionable editorial standards [8]. “Predatory journals,” as labeled by Beall (2012), provide a quick route to publication for a fee, but due to questionable editorial standards, may hurt the authors’ credibility long-term [9]. This phenomenon has also led to the conflation of OA and predatory journals as identical concepts, while in fact many OA journals have rigorous publication standards [10]. The prevalence of the conflation between predatory journals and open access at our institution remains unclear.

The components of open science, from open access [11] to data sharing [12] and open-source software [13], have been identified as critical components of science for over a decade. The adoption of open science practice has been encouraged, and in cases also mandated, by research funders around the world [[14], [15], [16], [17]].

At research institutions, incentives range from broad policies such at the UC Systemwide Academic Senate OA Policy to modified licensing agreements with publishers [18]. Modified license agreements enable universities to subsidize or completely cover article processing fees for researchers [18,19]. Despite these incentives, adoption of OA practices has been uneven [20]. Moreover, response to what is called a “reproducibility crisis” has highlighted the importance of comprehensive sharing of other research objects such as data, code and detailed methods underlying publications for certain disciplines beyond the finished manuscript [[21], [22], [23], [24], [25]]. More recent policies focus on transparency in research to address reproducibility challenges in research. These policies include the 2023 NIH Data Management and Sharing Policy [26], new policy guidance from the US White House Office of Science and Technology [27], and a new UC Research Data Policy [28].

Understanding the barriers faced by authors in the publication process is essential. There have been a number of surveys examining open science broadly [[29], [30], [31], [32], [33]], open access publishing [[34], [35], [36]], data sharing [[37], [38], [39], [40]], open peer review [41], open source [42], and the role of open science in research assessment [43,44]. These studies point to the methodological feasibility of a survey on open science and the likelihood of a satisfactory response rate. The studies were also successfully completed without putting respondents in any form of risk.

At our institution, we are closely watching new agreements with scientific publishers that represent a compromise between pay-to-publish models and standard subscription models. These agreements, dubbed “transformative agreements,” represent a top-down push for more openness within scientific publishing. Assessing those research outputs to gauge the efficacy of these new agreements is challenging. Presently, there is no central reporting system that tracks publications linked to the institution that could indicate immediate shifts in publishing habits as the result of new publishing agreements. Increased use of open science collaboration platforms like bioRxiv indicates enduring bottom-up interests in open science practice, but are even more difficult to track and record accurately [45].

Our investigation assessed the output format and publishing perceptions of science researchers at our institution in order to determine motivations for publishing in preferred venues and at various levels of open access and, more broadly, to sharing other research outputs. We also examined the involvement of researchers in the publishing process, from editorial service to promotion on social media. We hope to use this assessment to build more effective open access advocacy strategies. Disciplines investigated cover the physical sciences, life sciences, health sciences, engineering, and mathematics. Faculty, research staff, post-doctoral researchers and graduate students responded to questions through both a detailed online survey and in-depth interview. Questions addressed respondents’ opinions on and adoption of open science practice, including data publishing and preprint publishing. A pre-registration of this study was submitted to Open Science Framework [46].

While existing studies point to a methodologically sound, ethically completable, and scientifically insightful approaches, this study offers several novel opportunities. The first is a survey of a single university. This approach offers insights into research culture at a single location and provides useful data for future open science efforts on campus. The survey was expansively multi-disciplinary within the sciences. Researchers from across the life, physical, engineering, and health sciences, including the School of Medicine, were surveyed offering a nuanced picture of both the larger University research enterprise as well as distinct disciplinary research cultures. As a case study, examining the University of California, Los Angeles is particularly compelling since it is a public institution that shares similarities with public R1 institutions nationwide, and has strong consortial links to other University of California campuses. University of California campuses are well resourced and boast high enrollment – as such, they serve as a model for academic policies and spark global trends.

## 2 Materials and methods

### 2.1 Research design

Researchers approached gathering respondent data in two phases. The first phase involved a detailed online survey using the Qualtrics platform. At the end of this survey, respondents were asked if they would like to consent to an interview. The second phase involved interviewing consenting respondents for more details surrounding their scholarly publishing experiences. As this study involved minimal risk to human subjects, we received an IRB exemption from the UCLA Office of the Human Research Protection Program (IRB#21–000885).

Survey respondents were recruited via a campuswide email sent by the University Librarian, and direct outreach to faculty via departmental listservs between July 19, 2021 and August 3, 2021. Responses to the online survey were accepted through September 29, 2021. Follow up interviews occurred online via Zoom between August 30, 2021 and October 6, 2021. The survey could be viewed within a browser window or on a mobile device. Once submitted, responses could not be edited.

The survey was divided into four main sections. First, we gathered general information about the subjects’ departmental affiliation, academic status and years of experience measured by number of years post-Ph.D. Second, we asked questions about primary research outputs, publishing priorities, preferences and involvement with the publication process. Third, we gathered basic information about research information and data management practices. Lastly, we asked respondents how they and their respective departments tracked research outputs and impact. A redacted copy of the questions used for this study are available in Figure Supplement 1.

Online survey respondents who consented to and attended a scheduled interview were compensated with a $25 gift card to campus retailers. The one-on-one interview allowed researchers to push the respondents for publishing priorities and motivations, as well as recurring challenges and concerns. Interview questions centered scholar priorities but varied marginally depending on respondent status. For example, graduate students were asked how their mentor influences their publishing choices, and faculty were asked about their approach to mentoring trainees in choosing publications. The ability to ask follow-up questions about priorities and decision-making processes provided greater insights into the reasoning behind researcher publication choices. Two researchers were present at each online interview to serve distinct roles. One researcher conducted the interview with the respondent, asking questions and follow-ups, while the other research took detailed notes.

### 2.2 Survey analysis

Data analysis was performed using the R programming language and the following computational packages: for data cleaning and grouping we used ′dplyr' version 1.0.8, ′tibble' version 3.1.6, ′tidyr' version 1.1.4, ′reshape2′ version 1.4.4 and ′stringr' version 1.4.0. For data visualization and content organization ′ggplot2′ version 3.3.5, ′RColorBrewer' version 1.1–2 and ′knitr' version 1.36. Code and supplementary material are available on UCLA Dataverse [47].

Respondents were grouped based on career status or disciplinary area. For groups based on career status, groups were determined based on length of time since they completed their terminal degree, in the case of our data most respondents had completed or were in the process of completing their PhD. Individuals who had not yet completed or were less than 5 years post-PhD were grouped as “Early Career” researchers. Individuals between 6 and 25 years post-PhD were grouped as “Mid-Career” researchers. Individuals 26+ years post-PhD were grouped as “Full Career” researchers.

Groups based on disciplinary area were determined based on similarities in department structure, departmental incentives, and subject area. A detailed description on departmental groupings can be found in Figure Supplement 2. “Basic Sciences” departments include: “Biosciences,” “Chemistry,” “Public Health,” and “Institute for Environment and Sustainability.” Under “Pre-Professional” departments we included “Medicine,” “Dentistry,” “Nursing,” “Health Policy,” and “Psychology.” Under the category of “Physical Sciences” we included “Engineering,” “Physics,” “Atmospheric and Oceanic Sciences,” “Earth, Planetary and Space Sciences,” “Mathematics,” and “Computer Science.”

Comparisons between groupings for Yes/No/Unsure responses were assessed with Χ^2^ tests of independence or Fisher's exact test where Χ^2^ estimations were not accurate due to small group counts. For survey questions with multiple categorical responses, we used Χ^2^ test goodness of fit tests to test if proportions were equal between groups. Multiple comparisons were accounted for using a Bonferroni multiple comparisons approach.

### 2.3 Qualitative analysis

Free response questions were used to help respondents articulate more details about grant funding, primary publishing criteria, data publishing or sharing, descriptive information about assessment and reproducibility practices. Responses were reviewed in full to create coded categories for similar response types. Mentions of key words or relevant statements within respondent answers were tallied based on the created response types. Tallied responses were then grouped based on career status or department affiliation to illuminate patterns of responses.

## 3 Results

### 3.1 Population demographics and publishing preferences

**Fig. 1:**
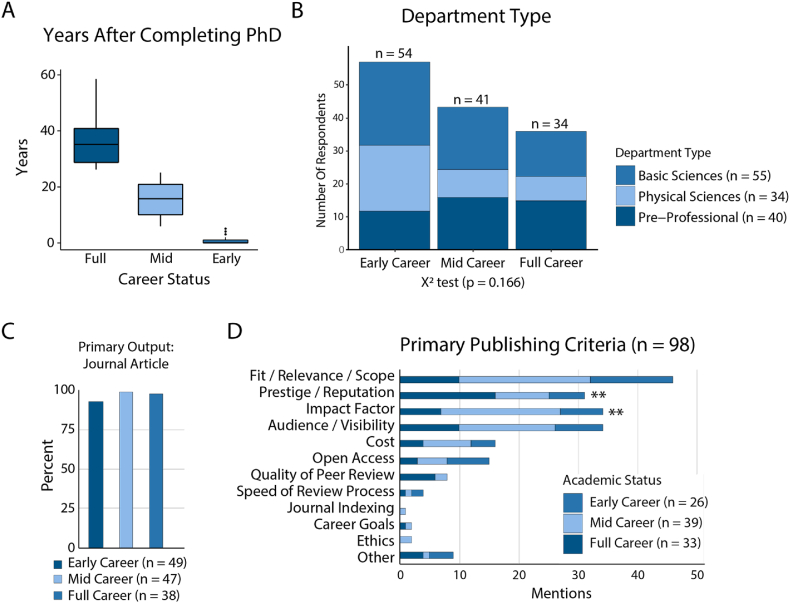
Survey Respondent Characteristics and Primary Publishing Criteria. A. Distribution of academic experience grouped by career status measured by years after completing PhD. B. Department type grouped by career status. C. Primary publishing outputs. D. Primary publishing criteria, free-response answers were binned based on similarity and grouped by academic status see Figure Supplement 2 for sample responses and binning strategy. **p < 0.005 using a χ^2^ goodness of fit test.

Survey respondents include a group of 182 researchers representing various academic statuses and departments. We hypothesized that individuals at different levels of their career might have different priorities, preferences, and practices for their publishing. Based on our sampling distributions we created three bins: early-career (less than 5 years post-PhD), mid-career (6–25 years post-PhD), and full-career (26+ years post PhD) researchers (Fig. 1A). We found that different career statuses were distributed evenly across our departmental groupings (Fig. 1B, Supplemental Fig. 2A). Our largest departmental representations were researchers in Biosciences, Medicine, Engineering and Public Health. We received responses from 15+ uniquely identifiable departments, however, several respondents did not indicate their departmental affiliation or described their affiliation as “Other” (Supplemental Fig. 2A).

We note that grouping by career status in this way, our sample sizes are greater than 30 for each group, giving us confidence that we can make statistical comparisons between the different groups (Fig. 1B). Using a Person's Χ^2^ test of independence we found that career status was not significantly dependent on departmental groupings (p = 0.166). We observe that Full career researchers are our smallest group, comprised of 34 respondents, mid-career researchers comprised 41 respondents and early-career researchers represent the largest group with 54 respondents. Most respondents (90.7%) indicated their primary research outputs were journal articles (Fig. 1C). The respondents who indicated some other output type indicated that they published book chapters, books, or conference papers.

Respondents were asked to identify attributes of publishing criteria such as fit, reputation, impact factor, or openness that represented the most important consideration to them when selecting publication venues. Responses were grouped by career status and binned based on similarity (Fig. 1D, Supplemental Fig. 3). Responses relating to the fit, relevance or scope were most frequent at 22.8% of all mentions. Impact factor and audience/visibility were the second and third most frequently mentioned criteria at 16.8%. Prestige/reputation was the fourth most mentioned at 15.3%. Cost was mentioned by 7.9% of respondents and open access was mentioned by 7.4% of all respondents.

Using Χ^2^ goodness of fit tests, we observe that the distribution of responses is not equal between groups, particularly regarding Prestige/Reputation (p = 0.0034) and Impact Factor (p = 0.0025). Notably, mid-career researchers were more than twice as likely to mention impact factor than any other group. In addition, full-career researchers were nearly 3 times more likely than early-career researchers to mention prestige or reputation as a primary criterion (Fig. 1D). Free responses in the survey indicated many respondents considered impact factor but for many it was only one of several factors considered in publishing decisions (Supplemental Fig. 3). Open Access was the 6th most mentioned criteria, for which we did not see significant differences between groups based on career status or department type.

### 3.2 ORCID use and preprint publishing

**Fig. 2:**
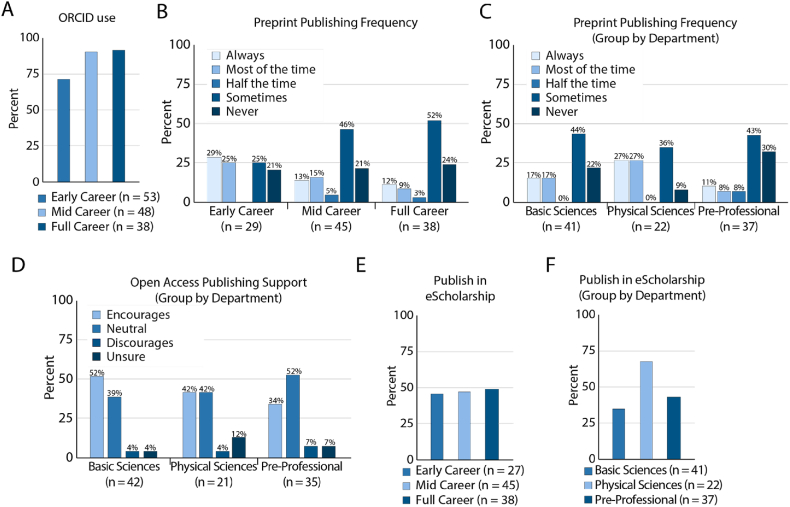
Publishing practices and participation among research grouped by career status. A. Percent of Respondents with ORCID IDs. B. Preprint publishing frequency among respondents grouped by career status. C. Preprint publishing frequency among respondents grouped by department type. D. Departmental support of open access publishing. E. Percent of respondents who publish manuscripts in eScholarship, or similar manuscript repositories grouped by career status. E. Percent of respondents who publish manuscripts in eScholarship, or similar manuscript repositories grouped by department type.

Individuals at different stages in their career participate in open access publishing and open research communication in different ways. Open Researchers and Contributor IDs, known as ORCIDs, are used among journals and publishers to connect researchers to their contributions. Academic libraries often see ORCID use as an entry point to open access publishing. We observed that a large majority of all researchers use ORCIDs (Fig. 2A). When grouped by career status, 94% of full-career researchers and 95% of mid-career researchers have ORCIDs and 70% of early-career researchers have ORCIDs, indicating a possible growth area for OA advocacy among early career researchers. We found ORCID use was dependent on career status (χ^2^ test: p < 0.001), but not dependent on department (χ^2^ test: p = 0.1202). (Fig. 2A). We note that a large number of common journals, regardless of whether or not they are open access, use ORCID identifiers. With such a large majority of our respondents using ORCIDs, it may be that this is not a sufficient metric to indicate open access publishing support.

Preprint publishing is a key way in which investigators can participate in open access publishing at low cost. When grouping by career status, we found that mid-career researchers and full-career researchers were appear more likely to sometimes, or never publish preprints (Fig. 2B). When grouping by department, we observe that physical sciences departments are most likely to publish preprints always or most of the time (Fig. 2C). Virtually all individuals asked, regardless of department type were neutral towards, or supportive of, publishing in open access journals and therefore saw no significant relationship between department-level open access publishing support and publishing of preprints (Fig. 2D).

The University of California system manages eScholarship a manuscript repository similar to preprint repositories such as arXiv and BioRxiv. When grouping by career status we see that all groups published in eSchoalrship at similar rates (Fig. 2E). When grouping by department we observed that researchers in physical sciences were more likely to publish in eScholarship or similar manuscript repositories than those with basic sciences department or pre-professional department affiliations (Fig. 2F). Using χ^2^ tests we found no significant dependence between eScholarship use and career status, or department. These data highlight that departments don't discourage their researchers from publishing in open access platforms but is consistent with what we observe in Fig. 1D in that open access publishing it is not a driving priority.

### 3.3 Barriers to open access publishing

**Fig. 3:**
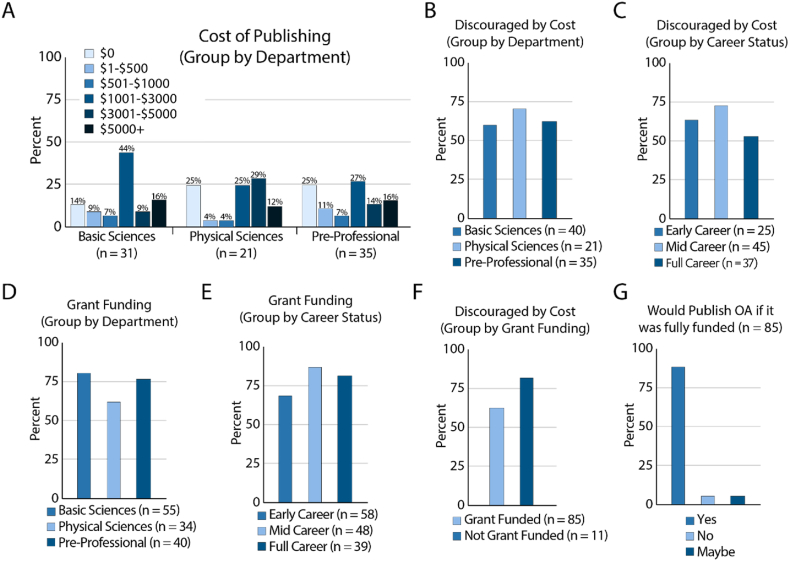
Publishing costs and barriers to open access funding. A. Cost of publishing grouped by department type. B. Discouraged by the cost of open access publishing grouped by department type. C. Discouraged by the cost of open access publishing grouped by career status. D. Grant funding grouped by department type. E. Grant funding grouped by career status. F. Discouraged by cost of open access funding grouped by grant funding. G. Open access publishing attitudes among grant funded researchers.

To estimate how much researchers spent on publishing we asked respondents to indicate how much they spend, on average, publishing in a peer reviewed journal in the last five years. We found that the cost of publishing is not equal among different department types (Fig. 3A). The majority of all respondents, regardless of career status or department are paying more than $1000 in that time frame. Regardless, we find that the majority of all respondents irrespective of department or career status are discouraged by cost of open access publishing (Fig. 3B and C).

To understand how research funding influences whether or not respondents are discouraged by the cost of open access publishing, we asked if they were supported by grant funding The majority of respondents, no matter the department or career status were supported by grant funding (Fig. 3D and E). When grouping by department we see that the proportion of labs that receive funding is not equal (χ^2^ test p = 0.0077), with fewer respondents in physical sciences supported by grant funding. No significant differences were seen based on career status. We found that grant funding status didn't significantly impact if respondents were discouraged by the cost of open access publishing (Fig. 3F). Notably, nearly all researchers said they would publish in Open Access journals if it was fully funded by an external source (Fig. 3G).

### 3.4 Data and information management practices

Data management and sharing of protocols, code, software and data have also grown substantially as a way to openly share research. We asked our respondents about their data management planning experience, their use of open-source software and publicly available data. In addition, we asked respondents who reported that they shared their own data where they choose to share data.

**Fig. 4:**
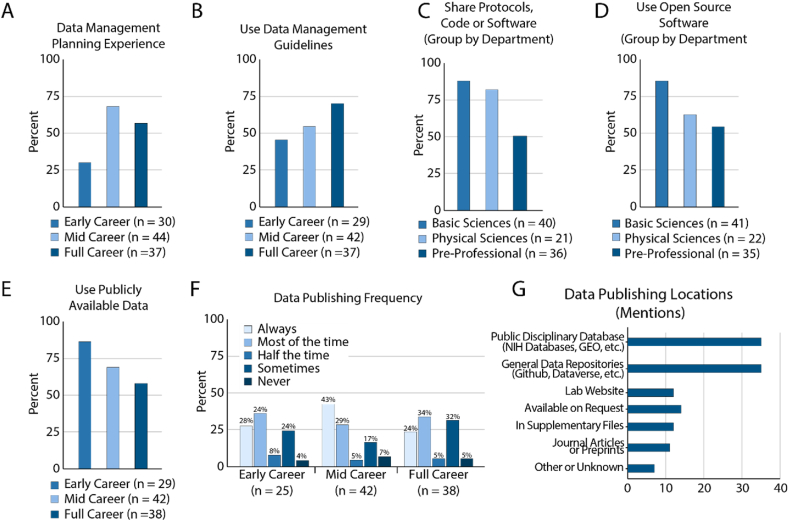
Data and information management practices. A. Respondents who have written a data management plan grouped by career status. B. Respondents who use or maintain data management guidelines in their research or laboratory settings grouped by career status. C. Respondents who share protocols, code or software grouped by department. D. Respondents who use open-source software as part of their work grouped by department. E. Use of publicly available data grouped by career status. F. Frequency of data sharing among respondents. G. Repositories or locations where respondents share data.

We found that participation in data management planning and setting data management guidelines is not equally distributed among respondents (Fig. 4A, χ^2^ test p = 0.0059). We see that early career researchers have the least experience (Fig. 4A and B), and that there are no differences between department types. We did see an unequal distribution between department types with regard to sharing protocols, code or software (Fig. 4C, χ^2^ test p = 0.00236) and using open-source software (Fig. 4D, χ^2^ test p = 0.0123). The majority of all respondents use open-source software or publicly available data, with early-career researchers reporting the highest proportion of use for publicly available data (Fig. 4D and E).

Data sharing generally refers to archiving of research data into disciplinary or general-purpose data repositories. The majority of all respondents claim to publish their research data always or most of the time regardless of career status or department (Fig. 4F). With our free responses we could get a better sense of what respondents meant when they say they share their data. A detailed description for how the responses were coded is included in Supplement 3. When asked where data is shared 28% mention disciplinary repositories and 28% mention general data repositories. Interestingly, 9.5% of the researchers we surveyed deposit data into their personal websites, and additional 11% of surveyed researchers share data on request (Fig. 4G). Together these data highlight that many of our respondents believe they are sharing data, but they don't always engage in practices that are considered open sharing of data.

### 3.5 Assessment practices by academic status

In addition to understanding the publishing priorities of our researchers, we sought to assess how researchers monitored their impact, and how their departments monitored their career progression. Citations and h-index are more traditional impact metrics focused on citations in other academic journals [48]. In contrast, altmetrics monitors a variety of online sources including blogs, social media and more to generate a score and can be considered a more inclusive metric for measuring impact used by researchers interested in open forms of scholarly communication like use of social media [49].

**Fig. 5:**
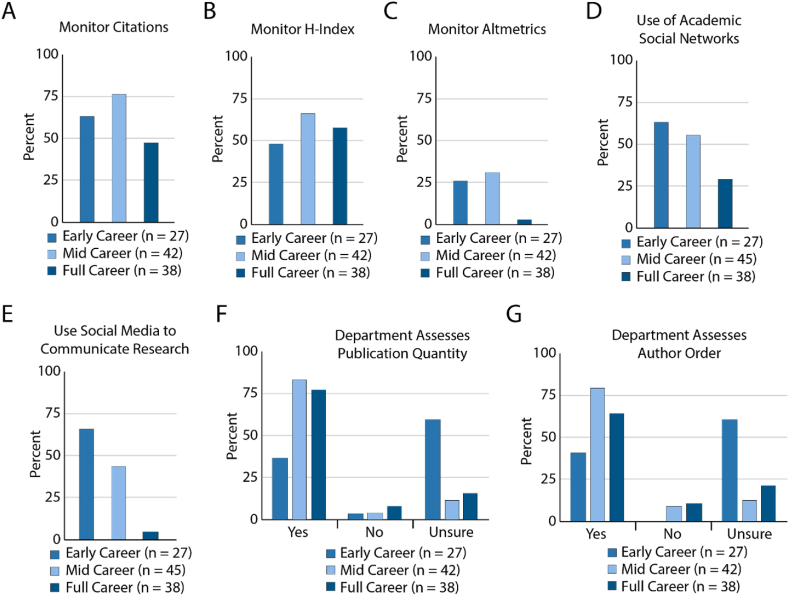
Assessment practices grouped by academic status. A. Percent of respondents who monitor their number of total citations for their work. B. Percent of respondents who monitor their H-index. C. Percent of respondents who monitor Altmetrics for their publications. D. Percent of respondents who use academic social networks such as ResearchGate or Academia.edu. E. Percent of respondents who use Twitter or other social media platforms to communicate about their research. F. Respondent awareness of departmental assessment of number of publications per individual for career advancement. G. Respondent awareness of departmental assessment of author order in publications for career advancement.

The majority of all respondents monitor their research impact by citation counts and h-index (Fig. 5A and B), though there are significant differences in who monitors citation counts (Fig. 5A, χ^2^ test p = 0.0057). Early and mid-career researchers are more likely to monitor impact using Altmetrics (Fig. 5C, χ^2^ test p = 0.00036). Early career and mid-career researchers are also more likely to communicate about their research in more open and public channels like academic social media networks such as ResearchGate and Academia.edu (Fig. 5D, χ^2^ test p = 0.0239) and popular social media platforms like Twitter (Fig. 5E, χ^2^ test p < 0.0001).

Methods of assessment that lead to promotion can be key motivating factors behind publishing decisions. Two common metrics often discussed include number of total publications which encourages collaboration, and author order which favors first or last author publications. Our results indicate that respondent awareness of assessment is not equal based on career status (Fig. 5F, χ^2^ test p = 0.0002 and Fig. 5G, χ^2^ test p = 0.0005). Early career researchers are most likely to be unsure if their departments measure impact in these ways, while mid and late career researchers are more likely to affirm that their departments measure impact in these ways (Fig. 5F and G), no significant differences were observed between department types and assessment practices.

Notably, free response questions in our survey revealed that authors felt that the pressure to monitor indications such as impact factor, prestige, visibility largely came from departmental assessment, or perceptions by which research impact is evaluated by granting agencies and their respective research community. Many respondents state this as matter of fact: “there's a noisy (but probably true) correlation between impact factor and career path.” Other investigators lamented this fact: “Unfortunately, the most important criteria are what others think of the journal …” Together these data highlight that early and mid-career researchers monitor their impact using more open platforms or inclusive metrics compared to full career faculty. However, departmental assessment creates a chilling effect on publishing in open access journals when they prioritize impact factor and prestige. Furthermore, since early career researchers are less sure of how they are evaluated, indicating a growth area for outreach and open access advocacy.

### 3.6 Interviews and advocacy strategies

Seventeen survey respondents consented to a follow-up interview. Of those seventeen interviewed, five were graduate students at various stages of their program, one participant identified as research staff, and eleven identified as professors. Of the professors, experience levels ranged from assistant adjunct to emeriti. Disciplinary representation was broad, however, the majority of interview respondents belonged to health and life science domains.

Our interviews added insights to respondents' selections. In our interviews with mid-career researchers, cost was the first criteria mentioned as a matter of concern. However, this was closely linked to journal prestige in surprising ways (Table 1). An early career Psychiatry respondent stated that “OA is rarely a consideration – if something is really exciting, we want to get it into larger journals.” Interviewers further contextualized that statement within the pre-tenure ecosystem of an incredibly competitive field. Access to research is complicated. Sure, a five-thousand-dollar article processing fee could make an article openly available, but as another Psychiatry department faculty pointed out, it could also fund an undergraduate researcher's summer in the lab. Given the choice, they would choose mentorship in the lab.

**Table 1:** Summary of key priorities, influences and advocacy strategies that emerged during one-on-one interviews. Strategies were grouped based on career status.

Career Status	Key Priorities	Key Influences	OA Advocacy Strategies
Early Career	•Promote openness •Sharing widely •Build publication record •Collaboration	•Advisor decision and goals •Altruistic views of access •Visibility of research	•Promoting preprint servers •Promoting data repositories •Highlighting ethical reasoning
Mid Career	•Increased impact •Career advancement •Minimize cost •Streamlined editorial process •Collaboration •Reproducibility	•Budgetary constraints •Departmental advancement •Journal reputation •Impact factors •Data publishing requirements •Speed of editorial process	•Reducing publishing cost to researcher •Promoting preprint servers •Promoting data repositories •Highlighting ease of collaborator access
Full Career	•Research visibility •Protecting data •Minimize cost •Speed of peer review •Reputation in discipline	•Familiarity with publications •Quality of editorial process •Data publishing requirements •Pre/post-print publication labor	•Reducing publishing cost to researcher •Articulating departmental merit standards •Promoting institutional openness goals •Promoting data sharing strategies

Survey free responses also indicate that researchers pay close attention to a journal's visibility and reputation in their field, and how publishing in a respective journal impacts their career advancement and grant competitiveness. A very small minority of full-career researchers surveyed prefer not to publish in open access journals, and conflate predatory journals with open access journals. Interviews provided some rationale for that choice. According to a senior professor in the Life Sciences, “affordable OA journals don't have great reputations because they're ‘fire hydrants,’” meaning they publish as much as they can without much editorial oversight. For some faculty, trust in the editorial process isn't there. A late career professor in the health sciences noted that some newer OA journals are not indexed by preferred databases. This indicates an opportunity for education as the vast majority of relevant OA journals are indexed in the most widely used databases in the health sciences, including Pubmed.

Regardless of discipline or experience level, researchers who collaborated directly with groups outside the academy highlighted the importance of access for their collaborators and stakeholders. Two separate public health researchers lamented problems with international co-author access to papers published in conventional subscription journals that otherwise met their criteria. An ecology graduate student expressed frustration in their ability to share published work with state and federal Fish and Wildlife stakeholders. Again, the selected publication had enough prestige, disciplinary fit, and editorial speed to move forward with publication. A Psychiatry faculty member said their work was hampered by an inability to share published work with local policymakers, but due to rigorous merit standards within the department, didn't feel they had much choice of journal selection.

Interviews revealed that consideration of impact factor is an ongoing discussion in many departments and is not prioritized uniformly across disciplines. A senior faculty member in Life Sciences commented, in response to impact factors, that “for years we didn't use metrics. Now we use them if they look good.” Senior faculty in Medicine stated that they advise trainees that “where you publish matters, but it can be overcome by citations. Eventually good work will find the light, but there might be a delay.” Unfortunately, in competitive fields, researchers may not have time to wait. One early-career respondent stated “several of my more recent papers have been in lower impact journals, and it's an embarrassment to me.” A respondent in Psychiatry said “my promotion coordinator will look down the list of publications, without reading them, and judge based on his perceived impact factor [of a publication]. Not the actual impact factor [of a publication].” Every researcher interviewed mentioned that there were niche journals specific to their field that were very important and trumped the value of more prestigious journals. Reasons for this varied. A respondent in Medicine claimed that “everything is available online, so as long as its on PubMed, it doesn't really matter.” Smaller subfields used the term “visibility” to highlight the validity of publishing in journals that may have a low impact factor but are very important to their specific discipline.

The overwhelming concern brought to our attention by respondents was the high cost of Open Access publishing. This response added nuance to our survey data, which listed cost as a priority, but less important than overall prestige. Surveyed respondents categorized their funding source as public (NSF, NIH), or university, meaning they have some responsibility to make their research public. Importantly, many respondents noted that their funding sources covered their research, but specifically not the dissemination. One researcher in the health sciences had been dissuaded from adding OA article processing fees to their grant application.

With regard to data publication and sharing, the most remarkable finding was that every single interview respondent had slightly different ideas about how and when it was appropriate to share their data. In the health sciences, researchers were most concerned about misinterpretation of data. In life sciences, researchers were concerned with other labs scooping their data before their lab had a chance to analyze multiple angles. In computational fields, data sharing was the norm and accompanied published manuscripts.

Graduate students interviewed seemed both acutely aware of challenges in the publishing space, and aware of their agency. While acknowledging stories of access challenges and a desire to share their work, they reported that all their publishing values were secondary to those of their supervisor. In ideal scenarios, they align.

## 4 Limitations

The chief difficulty in considering this study was the challenge of estimating an accurate study population. The campus is large, there is a porous border with a large healthcare system, and at any point faculty may be on sabbatical. Further, reaching all potential participants can only be confidently limited to Bruinpost (campus news) recipients since we could not confidently verify the number of researchers contacted by Student Affairs Offices. We overestimated potential respondents in our pre-registration, however, even assuming a much lower number of potential respondents, response numbers were low. Respondents were allowed to skip questions in the online survey, meaning some survey questions have even fewer responses.

This study is also subject to a response bias since researchers who did respond are demonstrating interest in the collective interests of their institution. They may exhibit a similar bias towards or against Open Access, the editorial process, and data sharing. It is challenging to generalize our findings beyond select participants who gave detailed responses given the small sample size and unknown bias of respondents. Statistical tests conducted on survey and free-response results are offered as exploratory findings and may have limited relevance to other institutions.

Our decision to group by discipline and career status made sense when considering UCLA's unique campus culture but may not be applicable to all institutions. Since departmental affiliation can be a fluid and imperfect way of grouping researchers, we sought to create groups with similar motivations and requirements unique to UCLA. We note that even though we expect there to be similarities based on our groupings, this study is not able to specifically determine that these groupings cause the differences we see and follow up studies need to be conducted to better understand causation behind our significant findings.

## 5 Discussion

Collectively, our data indicate that there are stark differences in priorities when comparing early-, mid- and full-career researchers. Even though all groups primarily publish journal articles as their primary research output, the priorities behind selecting where to publish varied in important ways. Findings from both the survey and interviews indicate that a researcher's publishing preferences rest in a delicate balance between the cost of publication and the desire to publish in a high-impact, branded journal (Fig. 1D and Table 1). Our results show a pattern where early-career researchers are more likely to want to participate in open science practices compared to their more experienced counterparts but have less agency over making publishing decisions that are open (Table 1). Opposing this are the priorities among mid-career researchers who are more concerned with cost and impact factor, and the priorities among full-career researchers who are more concerned with prestige.

The importance of impact factor and prestige was highlighted and justified by our findings, particularly for mid- and full-career faculty. Prestige was the most important factor among full-career faculty, more so than fit/relevance/scope (Fig. 1D). This is in alignment with comments from researchers indicating their publishing strategy starts with submitting to the highest-impact journal, even if it is a reach, and then gradually working their way to more discipline specific journals only if they are rejected (Interviews). Open access was a top-five priority only for early-career researchers. In the interviews, researchers would vocalize support of open access, then add a qualifier as to why it is not feasible. The most frequently mentioned concern was of cost, which was also reflected in our quantitative data (Fig. 3). According to our interviewees, it's not difficult to publish in an open access journal. However, the open access journals that are available at low cost are not sufficiently prestigious, and publishing in a branded or prestigious disciplinary journal is cost prohibitive. This suggests that transformative agreements need to be focused on journals with a high impact factor or based on their perceived reputation.

When reflecting on participation in publishing, we found it surprising that ORCID use was lower among early career researchers (Fig. 2B). This could be because early-career researchers may have not yet published and have not needed to create one. Interestingly, we found no relationship between career status or department type with regards to support for open access or publishing preprints (Fig. 2B–D). Researchers at all levels were unlikely to use eScholarship, a systemwide University of California institutional repository, as a venue for pre or post-print publication. This is likely because of a lack of visibility for manuscripts shared on this platform which was among the most mentioned respondent priorities (Fig. 1D).

Data management and publishing is a critical area of transformation in open science and many publishers and funders are focusing on data sharing to improve research transparency and reproducibility. The vast majority of survey respondents indicated that they publish data always or most of the time (Fig. 4F). However, a large proportion of respondents say they “Share data on request,” “share data on personal websites,” or share data in journal articles or supplements (Fig. 4G), which is not actually considered an open access practice. Interviews indicated that researchers view any level of sharing, even if it is “upon request, within subjective reason” as sharing. While this remains acceptable in certain fields, new policies such as the recent NIH policy on data management and sharing are beginning to define appropriate levels of sharing in order to amplify the transparency of research data [26].

Measuring research impact is a major challenge since it can take years or decades for research findings to influence policy or markets. Departments are best suited to assess the impact of their research, but there are several metrics such as citations, H-index, and Altmetrics that can be used to determine how the work is perceived by their respective fields. We find that early and mid-career researchers are more likely to monitor impact and publish about their work using more inclusive metrics and platforms. However, early career researchers remain more unsure about how they are assessed by their departments relative to their more experienced counterparts (Fig. 5F and G). This is an important training gap that, we discovered through our interviews, results in anxiety amongst trainees. In contrast, mid and full career faculty are more likely to be sure of how they are assessed which can discourage early career researchers from publishing in open platforms if they don't know it is an option for their own career growth.

As far as barriers to open access, most respondents indicate they are discouraged by cost irrespective of career status or department (Fig. 3B and C). Even though the majority of all respondents irrespective of career status or department are grant funded (Fig. 3D and E) they are still discouraged by cost of publishing. When we separate individuals who are and are not grant funded, the majority of respondents are still discouraged by the cost of Open Access (Fig. 3F). Free responses and interviews overwhelmingly indicate that cost is the most significant barrier to publishing OA (Fig. 3, Table 1). Importantly, if the cost was fully supported by the institution, 90% of respondents would be more likely to publish Open Access (Fig. 3G). Transformative agreements that aim to bolster open access publishing need to recognize that pushing article processing charges on to researchers will still discourage them from publishing in open access, even if they are grant funded.

In summary we find that cost and culture are the most substantial barriers to open access publishing. It is not surprising that advocacy strategies will need to vary based on career status, but our results offer insight into which strategies would be effective for researchers at different career stages. We find that early career researchers would benefit the most from learning about preprint and data publishing platforms and are likely to be receptive to ethical arguments for open access publishing. For mid-career researchers, finding ways to reduce the cost of publishing would be a direct way to increase OA publishing. Alternatively, encouraging preprint and data publishing as a means for increasing openness while still publishing in prestigious journals. This could be another way to increase openness in the publishing workflow as an alternative to paying for APCs in Gold Open Access journals. Finally, full career researchers may ultimately be the most difficult to reach since prestige or reputation of publishing venues is a driving force behind their publishing decisions. Ultimately, it may be that data publishing or open access publishing requirements may be the most effective way of enacting change for those who do not already prioritize it.

## Author contribution statement

Ibraheem Ali, M. Wynn Tranfield: Conceived and designed the experiments; Performed the experiments; Analyzed and interpreted the data; Contributed reagents, materials, analysis tools or data; Wrote the paper.

Jason Burton: Conceived and designed the experiments; Performed the experiments; Contributed reagents, materials, analysis tools or data; Wrote the paper.

## Data availability statement

Data associated with this study has been deposited at UCLA Dataverse https://doi.org/10.25346/S6/5OYU8Y.

## Additional information

Supplementary content related to this article has been published online at [URL].

## Declaration of competing interest

The authors declare that they have no known competing financial interests or personal relationships that could have appeared to influence the work reported in this paper.

## Appendix A Supplementary data

Supplement1 Supplement1

Supplement2 Supplement2

Supplement3 Supplement3
