# Supplementary material for: Assessing the publishing priorities and preferences among STEM researchers at a large R1 institution
Source: Heliyon. 2023 May 15;9(5):e16316. doi: 10.1016/j.heliyon.2023.e16316 (PMC10205490; doi:10.1016/j.heliyon.2023.e16316)
Supplement: Supplement1 [file mmc1.pdf]

Figure Supplement 1

Consent

The [redacted] division within [redacted] is conducting a research study intended to detail outputs of [redacted]. As an individual involved in the production of research products, your insights are highly valuable. Participation in this study will inform the future of [redacted] research services.

Online survey participation will take approximately twenty minutes to complete. Participants have the option to follow up with a short focus group, which will take no more than thirty minutes. If you volunteer to participate in this study, you will be asked:
- General questions detailing status at [redacted]
- Publishing preferences
- Data handling habits
- Research assessment practices

This study is being funded by the Librarians Association of the University of California, Los Angeles (LAUC-LA). Your participation in this research study is voluntary, and consent can be withdrawn at any point. If you have any questions, please contact [redacted] You may refuse to answer any questions that you do not want to answer and still remain in the study.

[redacted]

[redacted] Office of the Human Research Protection Program (OHRPP): If you have questions about your rights as a research subject, or you have concerns or suggestions and you want to talk to someone other than the researchers, you may contact [redacted]

- ○ Continue to survey
- ○ No thanks

Page Break

General Questions

Name

Department/Unit:

Status at UCLA:

- Faculty
- Post-Doc
- Graduate Student
- Undergraduate Student
- Staff

Highest Degree:

- Undergraduate
- Masters
- Ph.D

Total years post Ph.D (0 if student)

Is your work currently funded by Federal, State or Philanthropic grants?

- Yes
- No

If yes, please describe your primary funding source:

Page Break

Are you a member of a research lab at UCLA?

- Yes
- No

Do you have an ORCID ID?

- ○ Yes
- ○ No

If you would like to share it:

[ ]

Page Break

Do you have a professional/lab social media presence? If so, which platforms do you use?

[ ]

Page Break

Publishing

What is your most common research output?

- ○ Journal article
- ○ Book chapter
- ○ Other

If other, please describe:

[ ]

Page Break

Do you publish your work in preprint form?

- ○ Always
- ○ Most of the time
- ○ About half the time
- ○ Sometimes
- ○ Never

In your opinion, what are the most important criteria to consider when selecting a publishing venue?

Do you consider impact factors when selecting a journal to publish in? Why or why not?

How much have you paid, on average, for publishing in a peer-reviewed journal over the past five years? Include the cost of printing, colored figures, extra pages, APCs, etc.

- $0
- $1-$500
- $501-$1000
- $1001-$3000
- $3001-$5000
- $5000+

Has the cost of OA publishing discouraged you from publishing OA in the past five years?

- Yes
- No

In general, would you say your discipline encourages OA publishing, discourages OA publishing, or is neutral towards OA publishing?

- Encourages
- Discourages
- Neutral
- Unsure

Would you be more likely to publish OA if it was free or funded by an external source?

Yes or No Questions:

	Yes	No	Not sure
Do you consider your published work to be multidisciplinary?	○	○	○
Do you perform peer-review for journals in your discipline?	○	○	○
Do you participate as an editor for journals in your discipline?	○	○	○
Do you submit your work to OA repositories like eScholarship?	○	○	○
Do you share your work on sites like academia.edu or ResearchGate?	○	○	○
Do you share your work on Twitter?	○	○	○
Do you pre-register your studies?	○	○	○
Do you publicly share code, protocols, or software in your research?	○	○	○
Do you think there is value in publishing negative results?	○	○	○
Do you think there is value in publishing reproduced results?	○	○	○
Have you or would you publish in a journal that prints peer reviews alongside the article?	○	○	○

Page Break

Data

Have you written or assisted with a data management plan?

○ Yes

○ No

If so, did you utilize specialized tools like DMPTool when writing your plan? Explain.

Page Break

Yes or No questions about lab notebook, data and code management

	Yes	No	Not sure
Do you maintain a set of internal guidelines in your lab for managing and protecting data generated for your research?	○	○	○
Do you or does your lab utilize electronic lab notebooks?	○	○	○
Do you use open source software to perform data analysis tasks?	○	○	○
Do you utilize publicly available data in your published research?	○	○	○

Do you make finalized data associated with your publications publicly available?

- ○ Always
- ○ Most of the time
- ○ About half the time
- ○ Sometimes
- ○ Never

When you do make associated data publicly available, where do you publish it?

How do you and your team evaluate the reproducibility or interoperability of your work before sharing research outputs? For example, do you include a readme file or data dictionary that clearly describe variables and associated observations?

Page Break

Research Assessment

	Yes	No	Not sure
Do you track your own h-index	○	○	○
Do you track citations of your work?	○	○	○
Do you track altmetrics like retweets or shares?	○	○	○
Does your department consider publication quantity when considering career advancement?	○	○	○
Does your department consider first and/or last author designation in career advancement?	○	○	○

How does your department consider impact factors or journal prestige in career advancement?

Block 5

Would you like the opportunity to participate in a one-on-one interview? Participants consenting to one-on-one follow-up interviews will be eligible for $25 gift card compensation.

- ○ Yes, please consider me for a follow-up interview
- ○ No, thank you

Page Break

Please provide your best contact email:

Powered by Qualtrics
