# Supplementary material for: Assessing the publishing priorities and preferences among STEM researchers at a large R1 institution
Source: Heliyon. 2023 May 15;9(5):e16316. doi: 10.1016/j.heliyon.2023.e16316 (PMC10205490; doi:10.1016/j.heliyon.2023.e16316)
Supplement: Supplement2 [file mmc2.docx]

Assessing the publishing priorities and preferences among STEM researchers at a large R1 institution: Supplement 2

Ibraheem Ali, M. Wynn Tranfield, Jason Burton

2023-03-13

## ── Attaching packages ─────────────────────────────────────── tidyverse 1.3.2 ──
## ✔ ggplot2 3.4.1 ✔ purrr 1.0.1
## ✔ tibble 3.2.0 ✔ dplyr 1.1.0
## ✔ tidyr 1.3.0 ✔ stringr 1.5.0
## ✔ readr 2.1.3 ✔ forcats 0.5.2
## ── Conflicts ────────────────────────────────────────── tidyverse_conflicts() ──
## ✖ dplyr::filter() masks stats::filter()
## ✖ dplyr::lag() masks stats::lag()

## Respondent Characterization

Considering UCLA is a very large campus, departments at UCLA can vary in their funding support, their internal incentives, and overall size. In our self-reported departmental data we observed an uneven distribution of respondents at varying points in their career (Supplemental Figure 1A). We also observed that while some departments had a large number of respondents (15-30), other departments had very few respondents (2-4).


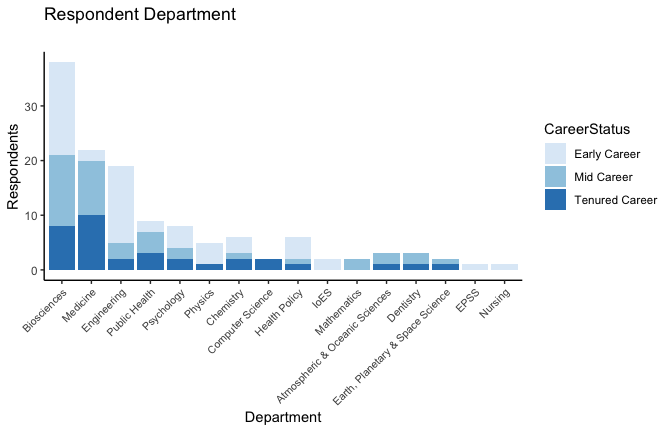


We conducted a chi-square test of independence to determine if there were significant differences in distribution of respondents at different career stages.

## Warning in chisq.test(department_independence): Chi-squared approximation may
## be incorrect

##
## Pearson's Chi-squared test
##
## data: department_independence
## X-squared = 47.557, df = 30, p-value = 0.02194

Based on the result of our Pearson’s Chi-squared test, we suspected that findings based characterized towards specific individual departments could be dependent on career status. Since several of our groups had sample sizes that were less than 4, we also recognize that our groupings needed to be larger in order to obtain meaningful results, as our Chi-square approximation when grouping individual departments was likely to be inaccurate.

To make our results easier to generalize, we created groups based on similarities in department structure, departmental incentives, and subject area. Briefly, groups in life and health sciences, including departments granting professional degrees, were separated from physical sciences due to differences in publishing behaviors observed by campus sciences librarians. We sought to further separate pre-professional departments from basic sciences departments because these departments do not typically require students to publish in order to complete their degrees (MD, RN, PsyD, DDS, etc.). Furthermore, many faculty are practicing clinicians, and are also less likely to rely on publishing for funding and credibility.

| Basic Sciences | Pre-Professional | Physical Sciences |
| --- | --- | --- |
| Biosciences | Medicine | Engineering |
| Chemistry | Dentistry | Physics |
| Public Health | Nursing | AOS* |
| IoES** | Health Policy | EPSS*** |
| Psychology | Mathematics |  |
|  | Computer Science |  |

* Atmospheric and Oceanic Sciences

** Institute of Environment and Sustainability

*** Earth, Planetary and Space Science

Using these groupings we were able to create larger bins with more even distributions of respondents by career status (Supplemental Figure 1B).


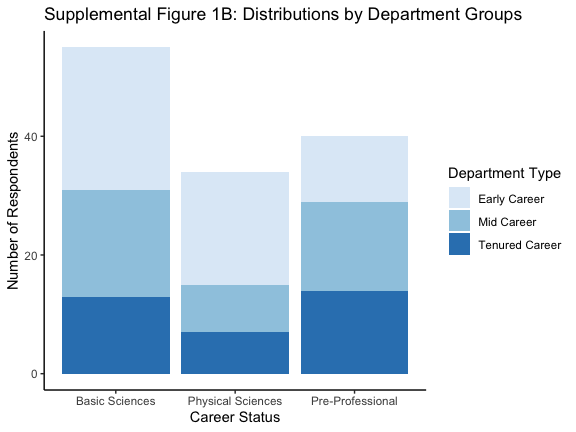


To ensure independence, we applied a chi-square test of independence to test if career status responses could be considered independently form department type.

##
## Basic Sciences Physical Sciences Pre-Professional
## Early Career 24 19 11
## Mid Career 18 8 15
## Tenured Career 13 7 14

##
## Pearson's Chi-squared test
##
## data: dep_car_test
## X-squared = 6.4819, df = 4, p-value = 0.1659

These results indicate that there is not sufficient evidence to show that our department groupings and career status groupings can be considered dependent on each other.
