# Supplementary material for: Assessing the publishing priorities and preferences among STEM researchers at a large R1 institution
Source: Heliyon. 2023 May 15;9(5):e16316. doi: 10.1016/j.heliyon.2023.e16316 (PMC10205490; doi:10.1016/j.heliyon.2023.e16316)

Assessing the publishing priorities and preferences among STEM researchers at a large R1 institution: Supplement 3

Ibraheem Ali, M. Wynn Tranfield, Jason Burton

2023-04-12

# Free Response Coding

## Assessing Publishing Priorities (Supplement 3A)

We sought to characterize publishing preferences and priorities for our respondents grouped by career status. We asked our respondents “In your opinion, what are the most important criteria to consider when selecting a publishing venue.” We used our career status coding to group responses based on their career status, below we report the sample sizes for each group.

## [1] "There were 26 Early Career responses"

## [1] "There were 39 Mid Career responses"

## [1] "There were 33 Full Career responses"

## [1] "There were 98 total responses"

We note a drop-off of early career researchers when responding to this question. Interviews with early career researchers (and some of their survey responses) indicate that early career researchers often do not feel as though their priorities or preferences matter relative to the preference of their faculty mentor.

Responses were hand coded and grouped for similarity. Key response types were determined after reviewing all free responses. In Supplemental Table 3 below we indicate the response type, and provide example responses for each grouping.

Certain respondents mentioned more than one response type. For these cases each comment was given one point per response type. For example in a response such as **“Impact factor, most fitting audience”**, this would receive one point for *Impact Factor*, and one point for *Audience/Visibility*. For responses with multiple key words describing the same response type such as **“Prestige, reputation, fit”**, they would receive one point for *Prestige/Reputation* and another point for *“Fit/Relevance/Scope”*, even though there were three coma-separated terms.

| Response Type | Example Response |
| --- | --- |
| Fit/Relevance/Scope | “Fit with the journal” |
| - | “Relevance to my field.” |
| - | “Compatibility in terms of theory, method and data” |
| Prestige/Reputation | “Prestige of outlet; turnaround time”* |
| - | “will people in my field see it and it is considered a good journal” |
| - | “Prestige of the journal and quality of editing and printing”* |
| Impact Factor | “Impact factor, relevance to my research field”* |
| - | “Impact factor, most fitting audience”* |
| - | “Impact factor, cost of submitting, fit of the journal with topic”* |
| Audience/Visibility | “Who will read it and how easily it can be found” |
| - | “Audience/readership of the journal, Impact factor”* |
| - | “audience” |
| Cost | “Low cost and open access”* |
| - | “Audience, reputation, publication fees”* |
| - | “Cost, audience”* |
| Open Access | “open access” |
| - | “Open access, but this decision is usually not mine to make” |
| - | “Open access, non-predatory journal* |
| Quality of Peer Review | “good quality peer review, journal is read well” |
| - | “scientific standing of the journal and review rigor”* |
| Speed of Reviewing Process | “speed of dissemination to the community…” |
| - | “timeliness of journal to publish papers after acceptance” |
| - | “fast review process” |
| Journal Indexing | “indexing and impact factor” |
| Career Goals | “The academic credit that I will get from publishing in that venue.” |
| Ethics | “…reputation/ethics of publisher.”* |
| Other | “value of the curves” |
| - | “Reputation or journal, editorial board…”* |
| - | “Research fitness.” |

*these responses would receive a point for at least one other response type.

## Table of Coded Data

| Response Type | Early Career | Mid Career | Full Career |
| --- | --- | --- | --- |
| Fit/Relevance/Scope | 14 | 22 | 10 |
| Prestige/Reputation | 6 | 9 | 16 |
| Impact Factor | 7 | 20 | 7 |
| Audience/Visibility | 8 | 16 | 10 |
| Cost | 4 | 8 | 4 |
| Open Access | 7 | 5 | 3 |
| Quality of Peer Review | 0 | 2 | 6 |
| Speed of Reviewing Process | 2 | 1 | 1 |
| Journal Indexing | 0 | 1 | 0 |
| Career Goals | 0 | 1 | 1 |
| Ethics | 0 | 2 | 0 |
| Other | 4 | 1 | 4 |

Category <- c("Fit/Relevance/Scope", "Prestige/Reputation", "Impact Factor/Impact",
 "Audience/Visibility", "Cost", "Open Access", "Quality of Peer Review",
 "Speed of Peer Review", "Journal Indexing", "Career Goals",
 "Ethics", "Other")
EarlyCareerP <- c(14, 6, 7, 8, 4, 7, 0, 2, 0, 0, 0, 4)
MidCareerP <- c(22, 9, 20, 16, 8, 5, 2, 1, 1, 1, 2, 1)
LateCareerP <- c(10, 16, 7, 10, 4, 3, 6, 1, 0, 1, 0, 4)
Index <- 1:12

PubPriorities <- data.frame(Category, EarlyCareerP, MidCareerP, LateCareerP, Index)

rm(EarlyCareerP, MidCareerP, LateCareerP)

PubPrioritiesPivot <- PubPriorities %>%
 pivot_longer(cols = c(EarlyCareerP, MidCareerP, LateCareerP),
 values_to = "Mentions") %>%
 mutate(CareerStatus =
 case_when(name == "EarlyCareerP" ~ "Early Career",
 name == "MidCareerP" ~ "Mid Career",
 name == "LateCareerP" ~ "Tenured Career")) %>%
 select(!name)

PubPrioritiesCompute <- PubPrioritiesPivot %>%
 mutate(Total = sum(Mentions)) %>%
 group_by(Category) %>%
 mutate(CountsByCategory = sum(Mentions))

PubPrioritiesCompute <- PubPrioritiesCompute %>%
 group_by(CareerStatus) %>%
 mutate(CountsByStatus = sum(Mentions))

PubPrioritiesCompute <- PubPrioritiesCompute %>%
 mutate(PercentByPriority = round(CountsByCategory / Total * 100, digits = 2)) %>%
 mutate(PercentByStatus = round(Mentions / CountsByStatus * 100, digits = 2))

ggplot(PubPrioritiesPivot, aes(y = reorder(Category, -Index),
 x = Mentions, fill = CareerStatus)) +
 geom_col()+
 theme_classic()+
 scale_fill_brewer(palette = "Blues")+
 labs(title = "Primary Publishing Priorities",
 x = "Mentions",
 y = "Priority",
 fill = "Career Status")


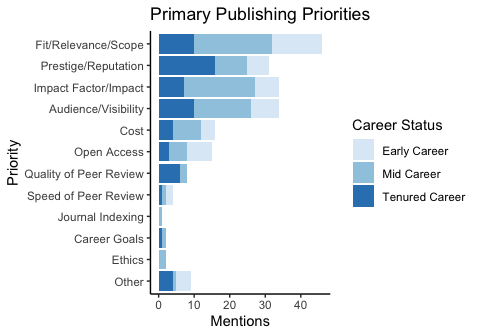
 To check to see if the overall proportion of responses were different from expected, we conducted a fisher exact test comparing response type frequencies with academic status frequency. We selected a fisher exact test instead of a chi-square test due to a low frequency of responses in the less-mentioned categories.

##
## Fisher's Exact Test for Count Data with simulated p-value (based on
## 2000 replicates)
##
## data: table(PubAssessTalies$ResponseType, PubAssessTalies$CareerStatus)
## p-value = 0.05097
## alternative hypothesis: two.sided

Our fisher exact test indicated that there is a relationship between the career status with the frequency of a given response. To assess the relationship between the frequency a respondent for a given academic status would mention a particular response type, we conducted a series of chi-square goodness of fit tests.

# Audience/Visibility

## Chi-squared test for given probabilities
##
## data: c(8, 10, 16)
## X-squared = 5.346, df = 2, p-value = 0.06904

# Cost

## Chi-squared test for given probabilities
##
## data: c(4, 4, 8)
## X-squared = 2.7684, df = 2, p-value = 0.2505

# Fit Relevance Scope

## Chi-squared test for given probabilities
##
## data: c(14, 10, 22)
## X-squared = 5.5318, df = 2, p-value = 0.06292

# Impact Factor / Impact

## Chi-squared test for given probabilities
##
## data: c(7, 7, 20)
## X-squared = 11.927, df = 2, p-value = 0.002571

# Open Access

## Chi-squared test for given probabilities
##
## data: c(7, 3, 5)
## X-squared = 0.32408, df = 2, p-value = 0.8504

# Other

## Chi-squared test for given probabilities
##
## data: c(6, 6, 6)
## X-squared = 0.6587, df = 2, p-value = 0.7194

# Prestige / Reputation

## Chi-squared test for given probabilities
##
## data: c(6, 16, 9)
## X-squared = 11.327, df = 2, p-value = 0.00347

# Quality of Peer Review

## Chi-squared test for given probabilities
##
## data: c(0, 6, 2)
## X-squared = 10.647, df = 2, p-value = 0.004876

Several groups were too small to generate accurate estimates. However, we found that two criteria were found to be significantly different from their expected proportions. These were Impact Factor / Impact (p = 0.002571) and Prestige / Reputation (p = 0.00347). For impact factor this is likely due to the high proportion of Mid-Career researchers that mentioned impact factor relative to early and full career researchers. For Prestige/Reputation, this is likely due to the high proportion of full career respondents who mentioned prestige/reputation relative to early and mid career researchers.

## Open Access if Free (Supplement 3B)

We asked respondents using a fill-in type response to indicate if they would be *more likely* to publish open access if it was free, or funded by an external source. We found three main categories of response, the first category yes, maybe and no. There were some responses that did not answer the question that were excluded. The table below shows example responses for each category:

| Response Type | Example Response |
| --- | --- |
| Yes | “YES!!!” |
| - | “Absolutely. I support open access strongly, but often can’t afford it” |
| - | “Yes, especially since I have no longer access to federal funds…” |
| Maybe | “Perhaps; I still prefer print journals” |
| - | “Yes, provided the OA review process met high standards” |
| - | “Maybe” |
| - | “Sure” |
| No | “No” |
| - | “no” |
| Excluded | “Not applicable” |
| - | “I have never published” |

### Counts grouped by response type

Early_Responses <- QualtricsDataCleaned %>%
 filter(OAifFree != "") %>%
 filter(CareerStatus == "Early Career")

Early_Responses$OAifFree

Mid_Responses <- QualtricsDataCleaned %>%
 filter(OAifFree != "") %>%
 filter(CareerStatus == "Mid Career")

Mid_Responses$OAifFree

Full_Responses <-QualtricsDataCleaned %>%
 filter(OAifFree != "") %>%
 filter(CareerStatus == "Tenured Career")

| Career Status | Yes | Maybe | No | Excluded |
| --- | --- | --- | --- | --- |
| Early Career | 21 | 1 | 0 | 1 |
| Mid Career | 34 | 1 | 1 | 0 |
| Full Career | 21 | 3 | 4 | 2 |
| Totals | 76 | 5 | 5 | 3 |

Percent <- c(76/86, 5/86, 5/86)
ResponseType <- c("Yes","Maybe","No")

OAifFree <- data.frame(Percent, ResponseType)

ggplot(OAifFree, aes(x = ResponseType, y = Percent, fill = ResponseType)) +
 geom_col() +
 scale_fill_brewer(palette = "Blues")


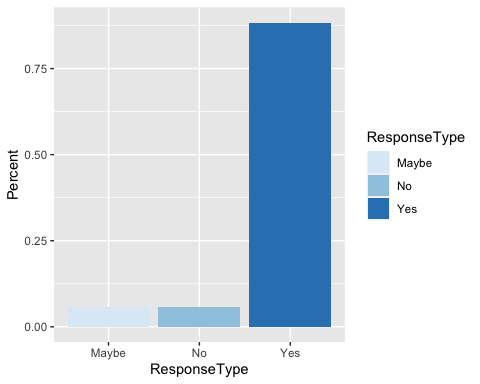


ggsave("OAifFree.pdf")

## Saving 5 x 4 in image

## Data Publishing Location Coding (Supplement 3C)

We asked respondents using a fill-in type response to indicate where they publish their data if they publish it alongside their manuscript. We found seven main categories of response outline below.

Certain respondents mentioned more than one response type. For these cases each comment was given one point per response type. For example in a response such as **“github, NCBI, ProteomeXchang”**, this would receive one point for *Public Disciplinary Database* (NCBI, ProteomeXchang), and one point for *General Data Repositories* (github).

The table below shows example responses for each category:

| Count | Response Type | Example Response |
| --- | --- | --- |
| 35 | Public Disciplinary Database | “NCBI database, github”* |
| - | - | “github, NCBI, ProteomeXchange” |
| - | - | “NIMH Data Archive” |
| 35 | General Data Repositories | “github / zenodo” |
| - | - | “GitHub” |
| - | - | “Supplement with article, GitHub, dryad” * |
| 12 | Lab Website | “… lab website” |
| - | - | “Website” |
| - | - | “on school website” |
| 14 | Available on Request | “by e mail on request” |
| - | - | “upon request” |
| - | - | “We provide it on request to individuals” |
| 12 | In Supplementary Files | “Technical appendix in the journal where the manuscript is published.” |
| - | - | “With the journal as supplemental material” |
| - | - | “GenBank or supplements to paper” |
| 11 | Journal Articles or Preprints | “These days we’re using OSF” |
| - | - | “Journals, genomic databases” |
| - | - | “upon submitting the preprint; Figshare” |
| 8 | Other or Unknown | “I have not yet published my own data” |
| - | - | “National Repository” |
| - | - | “self-hosted” |

ResponseTypes <- c("Public Disciplinary Database", "General Data Repositories", "Lab Website", "Available on Request", "In Supplementary Files", "Journal Articles or Preprints", "Other or Unknown")
Counts <- c(35, 35, 12, 14, 12, 11, 8)
Index <- 1:7

DataPubTypesPlot <- data.frame(ResponseTypes, Counts, Index)

ggplot(DataPubTypesPlot, aes(x = reorder(ResponseTypes, Index), y = Counts)) +
 geom_col(fill = "blue4") +
 theme(axis.text.x = element_text(angle = 90, hjust = 1))+
 labs(title = "Data Publishing Locations (Mentions)",
 x = "Response Type",
 y = "Mentions")


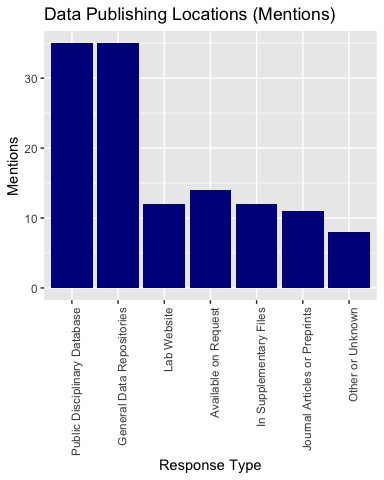

Supplement: Supplement3 [file mmc3.docx]
